# Robust pollution source parameter identification based on the artificial bee colony algorithm using a wireless sensor network

**DOI:** 10.1371/journal.pone.0232843

**Published:** 2020-05-15

**Authors:** MengLi Cao, Xiong Hu

**Affiliations:** Logistics Engineering College, Shanghai Maritime University, Shanghai, China; Newcastle University, UNITED KINGDOM

## Abstract

Pollution source parameter identification (PSPI) is significant for pollution control, since it can provide important information and save a lot of time for subsequent pollution elimination works. For solving the PSPI problem, a large number of pollution sensor nodes can be rapidly deployed to cover a large area and form a wireless sensor network (WSN). Based on the measurements of WSN, least-squares estimation methods can solve the PSPI problem by searching for the solution that minimize the sum of squared measurement noises. They are independent of the measurement noise distribution, i.e., robust to the noise distribution. To search for the least-squares solution, population-based parallel search techniques usually can overcome the premature convergence problem, which can stagnate the single-point search algorithm. In this paper, we adapt the relatively newly presented artificial bee colony (ABC) algorithm to solve the WSN-based PSPI problem and verifies its feasibility and robustness. Extensive simulation results show that the ABC and the particle swarm optimization (PSO) algorithm obtained similar identification results in the same simulation scenario. Moreover, the ABC and the PSO achieved much better performance than a traditionally used single-point search algorithm, i.e., the trust-region reflective algorithm.

## 1. Introduction

Hazardous pollution can be released to the atmosphere and cause severe disease to human beings [1]. At the early stage of the releasing process, the released pollution forms a plume near the pollution source. A timely perception of the released pollution and identification of its source parameters can save a lot of time for the subsequent elimination of the pollution source. Feasible solutions to this problem include deploying an array of stationary pollution sensor nodes [2] or dispatching mobile robots [3, 4] to the concerned area. The deployed sensor nodes can construct a WSN in a self-organized manner, which is capable of rapidly covering a large area.

The problem of PSPI based on a WSN has been extensively studied. With respect to the underlying theory, the WSN-based PSPI methods can be categorized to two groups: probabilistic methods and nonlinear least-squares (NLS) estimation methods. Probabilistic methods [5–7] output the estimated value with the highest probability of being the real value. Nehorai *et al*. [5] proposed a maximum likelihood estimation method to identify the source location, diffusion coefficient, and start time in a diffusion model. Keats *et al*. [6] proposed a maximum posterior probability method based on the Bayesian inference. Nofsinger *et al*. [7] proposed to implement the Ensemble Kalman Filtering algorithm to estimate the source location of a pollution plume. However, probabilistic methods usually assume that the measurement noises satisfy a specific distribution (e.g., the Gaussian distribution), which do not always hold in WSN-based PSPI applications.

Traditional NLS methods usually use single-point search algorithms to seek the solution that minimize the sum of squared measurement noises. Michaelides *et al*. [8] used the trust-region-reflective [9] (TRR) method to solve the NLS formulation of the WSN-based PSPI problem. Chen [10] proposed a *l*_2_-regularized NLS formulation of the WSN-based PSPI problem, and then solved it with a greedy method. The NLS formulation [11] does not require any statistical assumptions about the measurement noises, and thus, is robust to non-Gaussian measurement noises [12]. Nevertheless, when used for solving the NLS formulation of the WSN-based PSPI problem, traditional single-point search methods are prone to be stagnated by the premature convergence problem, i.e., converge prematurely at solutions far from the true-values of the parameters. This problem can be avoided by using the population-based parallel search approaches, e.g., the particle swarm optimization (PSO) algorithm [13]. As a relatively new population-based parallel search algorithm, the artificial bee colony (ABC) algorithm presented by Karaboga et al. [14] has been widely applied in various applications. A comprehensive survey of the ABC and its applications can be found in [15]. Compared with other population-based algorithms, the ABC can achieve better or similar performance with the advantage of employing fewer control parameters [16]. Moreover, the ABC has been found to be able to produce very good results at a low computational cost [15], which is significant for saving time in the PSPI processes. In the area of WSN, the ABC has been implemented for node localization [17], node clustering [18], network routing [19], coverage optimization [20], and etc. However, to our knowledge, the ABC has not been adapted for solving the WSN-based PSPI problem yet.

In this paper, we adapt the ABC to solve the problem of WSN-based PSPI. To identify the real value of the unknown parameters, i.e., the source location, release rate, and the diffusion coefficient, an objective function of them is formed based on the NLS paradigm. Then, the ABC is used to search for the global minimum of the objective function. To evaluate the ABC, it was compared with the PSO [21], the Levenberg-Marquardt (LM) [22], and the TRR in extensive simulations. Simulation results show that the ABC and the PSO achieved similar estimation results in the same simulation scenario. Moreover, they both can achieve much better performance than traditional single-point search methods. This paper includes at least three contributions: (1) presents a method for adapting the ABC to solve the WSN-based PSPI problem, and verifies its feasibility and robustness; (2) verifies that the ABC outperforms the traditionally used single-point search methods; (3) reveals the influence of algorithm parameters on the identification performance of all tested algorithms.

The rest of this paper is organized as follows. Section 2 describes the pollution distribution model and concentration measurements of the WSN, and the scheme of all simulations. Section 3 details the ABC, PSO, LM, and TRR algorithms, Section 4 presents the simulation results and corresponding discussions. Section 5 concludes the whole paper and gives some future works.

## 2. Simulation setup

### 2.1 Pollution distribution and measurement

Within an 80-by-80 m^2^ flat square area, we simulate a pollution source, which is releasing hazardous materials at a rate of *q* mL/s on location (*x*_s_, *y*_s_), i.e., the release rate is *q* mL/s. The released materials are carried by an isotropic wind at a speed of *v* cm/s, forming a time-averaged pollution distribution. At the meantime, *N* sensor nodes are randomly deployed in the area at locations (*x*_s_, *y*_s_), *i* = 1,…, *N*. The sensor nodes are capable of forming a WSN in a self-organized manner, and measuring its surrounding pollution concentration, which is denoted as *z*_*i*_, *i* = 1,…, *N*.

In real PSPI applications, the concentration measurements of the sensor nodes (i.e., *z*_*i*_, *i* = 1,…, *N*) are available. However, in computer simulations, the value of *z*_*i*_, *i* = 1,…, *N* should be obtained by adding an measurement error term *e*_*i*_, *i* = 1,…, *N* to the theoretical concentration *c*_*i*_, *i* = 1,…, *N*, as follows.

$$z_{i}=c_{i}+e_{i},i=1,\ldots ,N,$$ (1)

where *c*_*i*_, *i* = 1,…, *N* can be calculated by substituting $\left(x_{i},y_{i}\right),i=1,\ldots ,N$ into a pollution concentration distribution model. Considering the fact that real pollution sensors are constrained by the detection range, the *i*-th sensor node is hibernated if *z*_*i*_ < *lb* or *z*_*i*_ > *ub*, where *lb* and *ub* are the lower and upper bounds of the sensor detection range, respectively.

To calculate *c*_*i*_, *i* = 1,…, *N*, we adopt the advection-diffusion model presented in [23], which has been widely used to describe the time-averaged concentration distribution in environments with homogenous airflow [24–27]. It takes the form

$$c_{i}(x_{s},y_{s},q,K)=\frac{q}{2\pi K}\frac{1}{d_{i}}\exp\left[-\frac{v\left(d_{i}-x_{i}+x_{s}\right)}{2K}\right],$$ (2)

where *K* is the turbulent diffusion coefficient, *d*_*i*_ is the distance between $\left(x_{i},y_{i}\right),i=1,\ldots ,N$ and (*x*_s_, *y*_s_). In our concerned WSN-based PSPI problem, $\left(x_{s},y_{s}\right)$, *q*, and *K* in Eq (2) are parameters to be identified, since their real values are previously unknown in the real PSPI process. However, to simulate the measurement process and generate the concentration measurements, the real values of (*x*_s_, *y*_s_), *q*, and *K* should be substitute into Eq (2) to calculate *c*_*i*_, *i* = 1,…, *N*, and in turn calculate *z*_*i*_, *i* = 1,…, *N* based on Eq (1).

### 2.2 Simulation scheme

To solve the PSPI problem, we follow the NLS estimation paradigm [11] to simultaneously identify the value of $\left(x_{s},y_{s}\right)$, *q*, and *K*. For ease of expression, the four unknown parameters $\left(x_{s},y_{s}\right)$, *q*, and *K* are integrated in a four-dimensional vector $p=\left[x_{s},y_{s},q,K\right]$. Then, the NLS estimation problem [11] can be represented as searching the minimum of

$$obj(p)=\sum_{i=1}^{N}{\left[z_{i}-c_{i}(p)\right]}^{2}.$$ (3)

By considering the value of *fit* (***p***) = 1/obj (***p***) as the fitness of ***p***, the NLS estimation problem becomes an optimization problem. According the Eq (3), the value of *obj* (***p***) is positive. Thus, a solution that maximize the reciprocal of *obj* (***p***) (i.e., *fit* (***p***) = 1/obj (***p***)) can minimize *obj* (***p***).

In order to solve the PSPI problem, the ABC is used to maximize the value of *fit* (***p***) = 1/*obj* (***p***). The influence of ABC’s tunable parameters on the estimation performance is assessed. Then, to comprehensively evaluate the ABC, it was compared with three counterpart algorithms: the PSO, LM, and TRR algorithms, in extensive simulations.

Last but not the least, the robustness in respect of non-Gaussian measurement noises were evaluated. Typically, the measurement noises satisfy Gaussian distribution, whereas measurement noises satisfying non-Gaussian distribution [12] also exist in many real PSPI applications. Thus, to test and compare their robustness in respect of non-Gaussian measurement noises, the ABC and the PSO were conducted based on a series of concentration measurements with contaminated Gaussian noises, which can be represented as a convex combination of a Gaussian noise and a uniform noise, as follows [12].

$$e_{i}\sim \lambda N(0,10)+(1-\lambda )U(0,10),\lambda \in [0,1].$$ (4)

## 3. Search algorithms

### 3.1 Artificial bee colony

**Fig 1:**
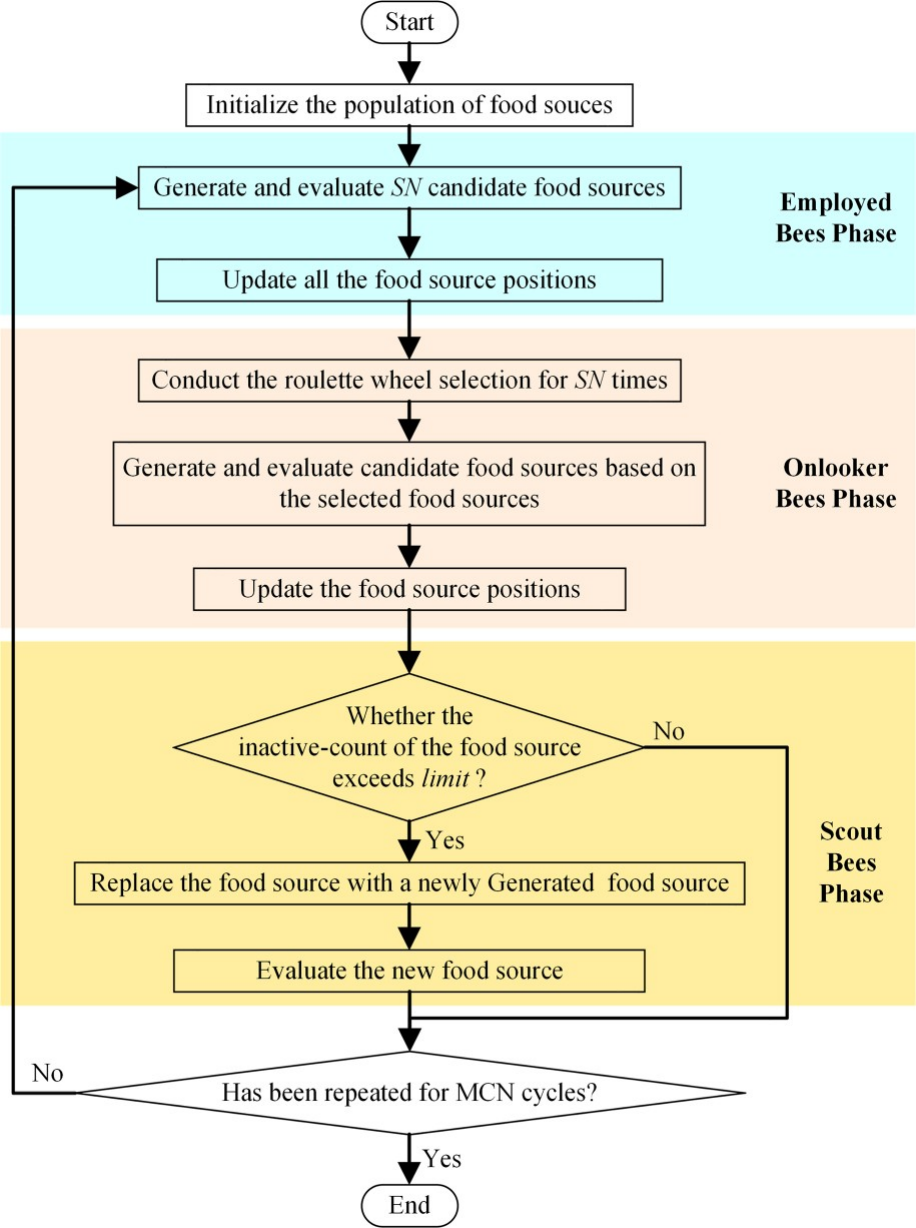
The inner logic flowchart of the ABC.

The inner structure of the ABC [15] is shown in Fig 1.

In the ABC, a solution and its corresponding fitness value are called a food source and its corresponding nectar volume, respectively. For the optimization problem stated in section 2, a food source takes the form of the vector ***p*** in Eq (3). The nectar volume of a food source is calculated by substituting ***p*** into Eq (3). At the beginning, the ABC enters the initialization phase, a population of *N* food sources are randomly generated within the valid value ranges. Then, the ABC algorithm enters a cyclic state. Each cycle consists of the employed bees phase, the onlooker bees phase, the scout bees phase, and a phase for updating the best solution achieved so far.

In the employed bees phase, a new candidate food source ${p^{\prime }}_{i}$ is generated by varying a randomly selected dimension of each food source in the population, as follows.

$$\begin{array}{l} {p^{\prime }}_{ij}=p_{ij}+\phi_{ij}(p_{ij}-p_{kj})\\ i=1,2,\ldots ,N,\;j\in \{1,2,3,4\}, \end{array}$$ (5)

where *ϕ*_ij_ is a uniform random number in the range [−1,1], *k* is randomly selected from the set [1,i−1]∪[i+1,N], $p_{ij}$ (${p^{\prime }}_{ij}$ or $p_{kj}$) denotes the *j*-th element of $p_{i}$ (${p^{\prime }}_{i}$ or $p_{k}$). Then, the nectar volume of ${x^{\prime }}_{ij}$ is evaluated. ***p***_*i*_ will be replaced by ${p^{\prime }}_{i}$ in case that $obj({p^{\prime }}_{i})<obj(p_{i})$. Otherwise, the inactive-count of ***p***_*i*_, which is denoted as *l*_*i*_, will be self-increased to *l*_*i*_ +1. The operations in the employed bees phase is inspired by the phenomenon that employed bees search for new food sources containing more nectar within the neighborhood of the food source in the memory [15].

In the onlooker bees phase, for each of the onlooker bees, a roulette wheel selection operation is conducted to select within the ABC population. Then, for the selected food source, a new candidate food source is generated and evaluated is conducted in the way like that in the employed bees phase. The operations in the onlooker bees phase is inspired by the phenomenon that onlooker bees probabilistically choose their food sources depending on the information provided by the employed bees [15].

In the scout bees phase, if the inactive-count of a food source exceeds a pre-defined “*limit*”, it will be replaced by a randomly generated food source. This operation is inspired by the phenomenon that scout bees randomly search for new food sources to replace the initially poor or fully exploited food source [15].

Finally, as the best solution achieved so far, the food source with the biggest fitness value in the population is updated.

### 3.2 Particle swarm optimization

**Fig 2:**
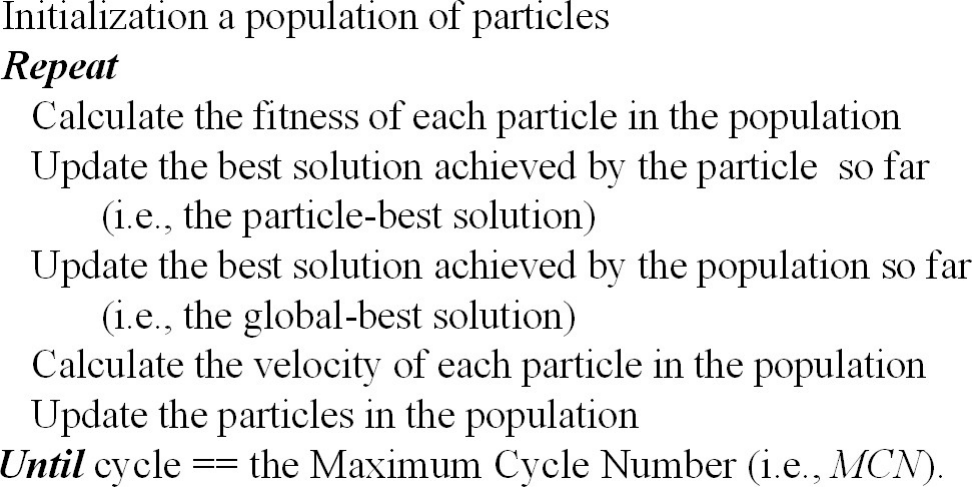
General structure of the PSO.

The general structure of the PSO [21] is shown in Fig 2.

For using the PSO to minimize *obj* (***p***) in Eq (3), the particle (i.e., the solution in the PSO) takes the form of ***p***. After the PSO solutions are randomly initialized within the valid value ranges, all of the particles are cyclically updated.

In each cycle, the fitness of all the particles are calculated. Then, the historically best solutions (i.e., the particle-best solution ***pb***_*i*_, *i* = 1,2,…,*N* and the global-best solution ***gb***) achieved by the particle and the population are updated, respectively. Then, the *i*-th particle is updated as follow.

$$\begin{array}{l} v_{ij}^{k}=\omega v_{ij}^{k-1}+\varphi_{1}r_{1}(pb_{ij}^{k-1}-p_{ij}^{k-1})+\varphi_{2}r_{2}(gb^{k-1}-p_{ij}^{k-1})\\ p_{ij}^{k}=p_{ij}^{k-1}+v_{ij}^{k}\\ i=1,2,\ldots ,N,\;j=1,2,3,4 \end{array},$$ (6)

where ***v***_*i*_ is the velocity of the *i*-th particle, *N* is the number of particles, *k* denotes the cycle number, the second subscript *j* is the dimension index (i.e., $v_{ij}^{k}$ is the *j*-th dimension of ***v***_*i*_ at the *k*-th cycle), *ω* is a nonnegative inertial factor, *r*_1_ and *r*_2_ are two random number within the range (0,1), *φ*_1_ and *φ*_2_ are acceleration constants. [21]

### 3.3 Single-point search algorithms

The LM method and the TRR method are two single-point search methods that are traditionally used for solving the NLS problem. Both methods have the common point that only one single solution is cyclically evaluated and improved.

For the NLS problem of minimizing ${\left\|F(p)\right\|}_{2}^{2}$ by varying the value of ***p***, the search direction of the LM method at the *k*-th cycle, denoted by ***d***^*k*^, satisfies [22]

$$\left(J^{T}(p^{k})J(p^{k})+\mu^{k}I\right)d^{k}=-J^{T}(p^{k})F(p^{k}),$$ (7)

where *J* (***p***) is the *Jacobean* matrix of *F* (***p***), *μ*^*k*^ is a constant coefficient. It is readily seen that, for the NLS problem concerned by this paper, F(p)=z−c(p).

The TRR method approximates the function obj(p) to be optimized by a much simpler function *q*(***p***) in the neighborhood of the current solution (i.e., the trusted region TR), and then solve the minimization problem [9]

$$\underset{p}{\min}\{q(p),p\in TR\}.$$ (8)

To determine the trusted region, the TRR method employs a preconditioned conjugate gradient process [28].

## 4. Results and discussion

As mentioned in section 2.2, a large number of simulations were conducted to tune the parameters of the ABC, PSO, LM, TRR methods, and then to compare their performance on solving the WSN-based PSPI problem.

### 4.1 Control parameter selection

A typical simulation scenario is generated to select the value of control parameters, which can influence the estimation errors. The real value of ***p*** is set as [1000 cm, 4000 cm, 8000 mL/s, 5000 cm^2^/s]. To generate the initial populations for the ABC and the PSO, the valid value ranges were empirically set as follows. The lower bound of *x*_*s*_, *y*_*s*_, *q*, *K* were set as 0 cm, 0 cm, 800 mL/s, 4000 cm^2^/s, respectively. The upper bound of *x*_*s*_, *y*_*s*_, *q*, *K* were set as 8000 cm, 8000 cm, 16000 mL/s, 6000 cm^2^/s, respectively.

The wind velocity, which can be measured in real applications, was set as 3 m/s. In order to keep enough sensor nodes at work, the value of *lb*, and *ub* were set as 50 ppm and 1000 ppm, respectively. The concentration measurement noises were sampled from the Gaussian distribution *N* (0,10).

#### 4.1.1 The ABC algorithm

In the ABC, there are three control parameters: the number of food sources *SN*, *limit* in the scout bees phase, and the maximum cycle number (*MCN*). *SN* is also the population size of the ABC. Empirically, like other population-based parallel-search methods, larger population size means higher search efficiency, while larger value of *MCN* means longer and more sufficient search. However, the increasing of *SN* and *MCN* both bring about computational cost. The computational complexity of the ABC, which is not the main concern of this paper, has been studied in [15]. Therefore, considering the computational resource of our experimental computer (i.e., Intel i7-7500U CPU and 8GB RAM), the value of *SN* and *MCN* was set as 100 and 500, respectively.

**Fig 3:**
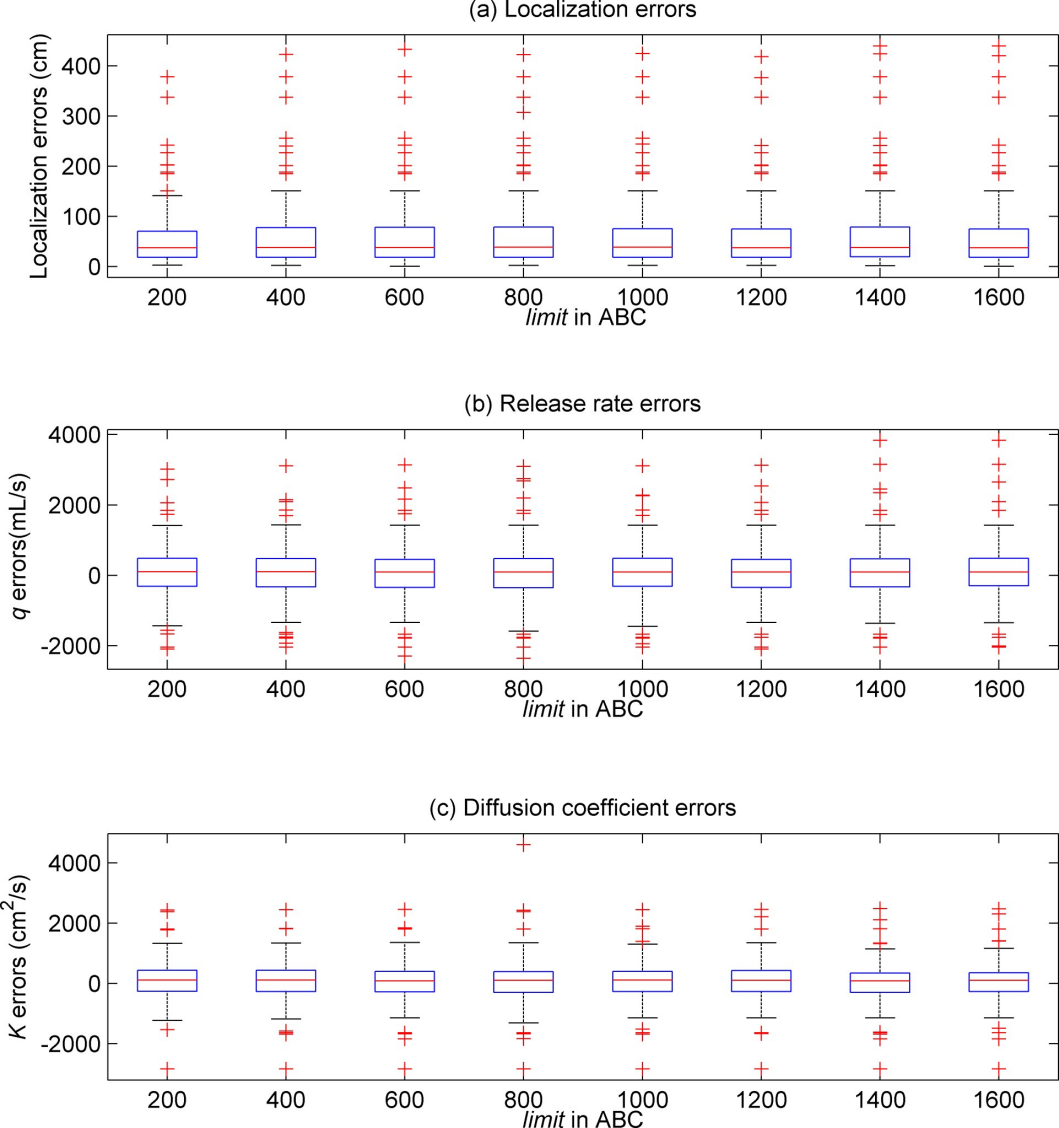
Boxplots of the estimation errors in eight groups of the simulation trials with different values of “*limit*” in the ABC.

To evaluate the influence of *limit* in our problem, the WSN was randomly deployed for 100 times. For each of the random node deployment schemes, the value of *limit* was set from 200 to 1600 in interval of 200. Boxplots of the estimation errors with respect to different *limit* values are shown in Fig 3. According to Fig 3, the distinctions between the results obtained with different *limit* values are minor. In all the six groups of trials, the medians of the estimation errors are close to zero. It seems that, for solving the WSN-based PSPI problem, the scout bees phase can hardly influence the search ability of the ABC. This is probably because the exploration strengths in the employed bees phase and the onlooker bees phase of the ABC are strong enough to maintain the ABC population diversity in our problem.

#### 4.1.2 Counterpart algorithms

The PSO, LM, and TRR algorithms are selected as the counterpart algorithms, which can also be used to solve the WSN-based PSPI problem concerned in this paper, to be compared with the ABC. It is necessary to tune the parameters of the counterpart algorithms too.

**Fig 4:**
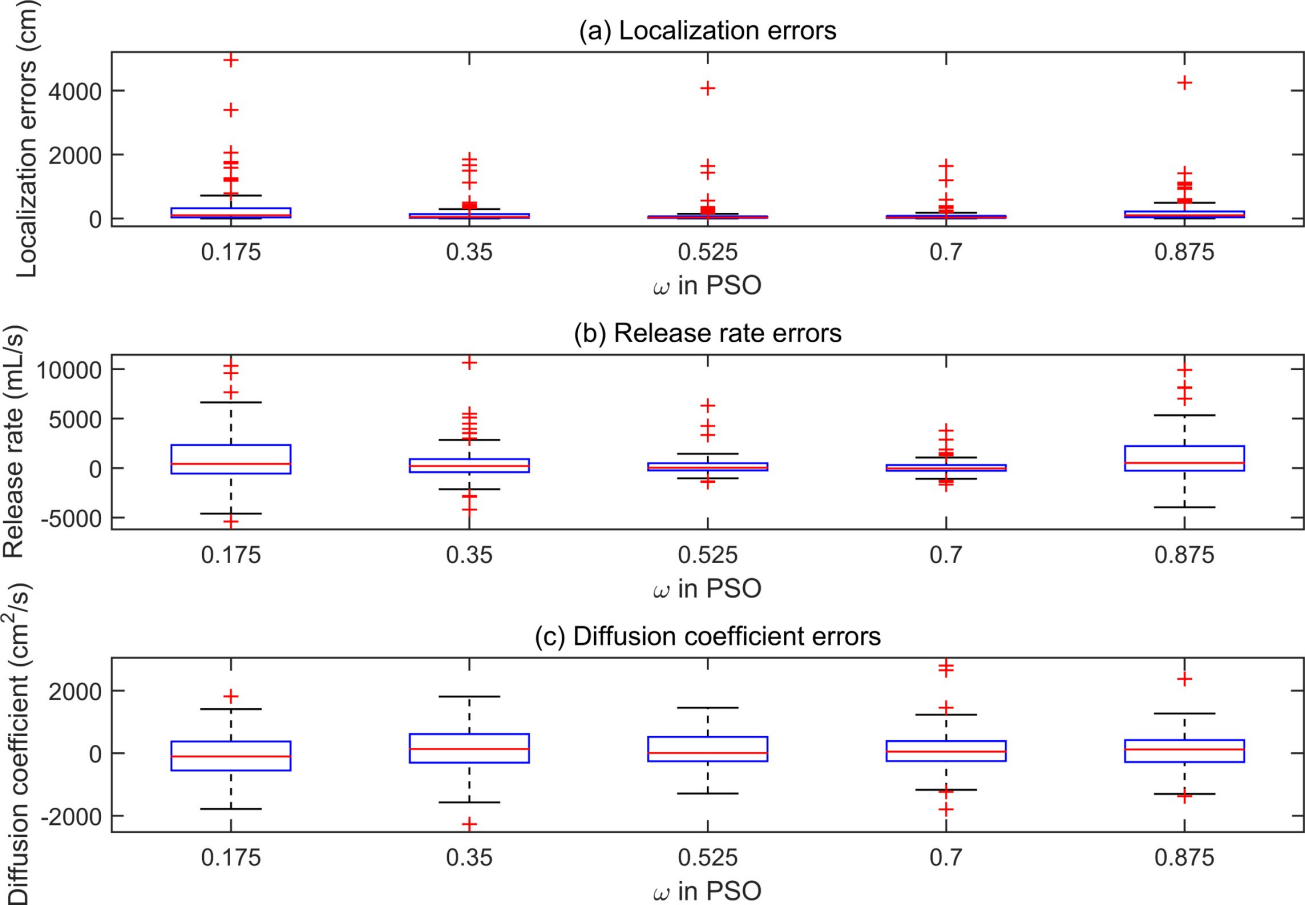
Boxplots of the estimation errors in five groups of simulation trials with different values of inertial factor in the PSO.

For rigorous comparison, the population size and MCN of the PSO were set as the same as those selected for the ABC, i.e., *N* = 100, *MCN* = 500. Moreover, there are three control parameters: the acceleration constants *φ*_1_ and *φ*_2_, and the inertial factor ω. Empirically, setting the value of the acceleration constants to 2, i.e., *φ*_1_ = *φ*_2_ = 2, can achieve good performance [21]. Thus, only the value of inertial factor *ω* was tuned. To evaluate the influence of inertial factor *ω* in our problem, the WSN was randomly deployed for 100 times. For each of the random node deployment schemes, the value of *ω* was set from 0.175 to 0.875 in interval of 0.175. Boxplot of the estimation errors in the five groups are shown in Fig 4.

As shown in Fig 4, when the value of *ω* equals 0.7, the *IQR* of the boxplot is smallest, and that the median of the boxplot is closest to zero. Thus, it can be concluded that the PSO obtained generally smaller estimation errors than in other cases. The most possible reason is that the small value of *ω* brings about small diversity of the particles.

For the two typical single-point search algorithms, i.e., the LM and the TRR algorithms, the influence of start point on the estimation performance was evaluated. Compared with the real value to be estimated, i.e., [1000 cm, 4000 cm, 8000 mL/s, 5000 cm^2^/s], the four start points in Table 1 are obviously different. The fourth start point is closest to the real values, whereas the first one has the biggest gap with the real values.

**Table 1:** Start points for LM and TRR.

ID	*x*_s_ (cm)	*y*_s_ (cm)	*q* (mL/s)	*K* (cm^2^/s)
1	4000	4000	2000	1000
2	4000	4000	2000	2500
3	2000	4000	4000	1000
4	2000	4000	4000	2500

**Fig 5:**
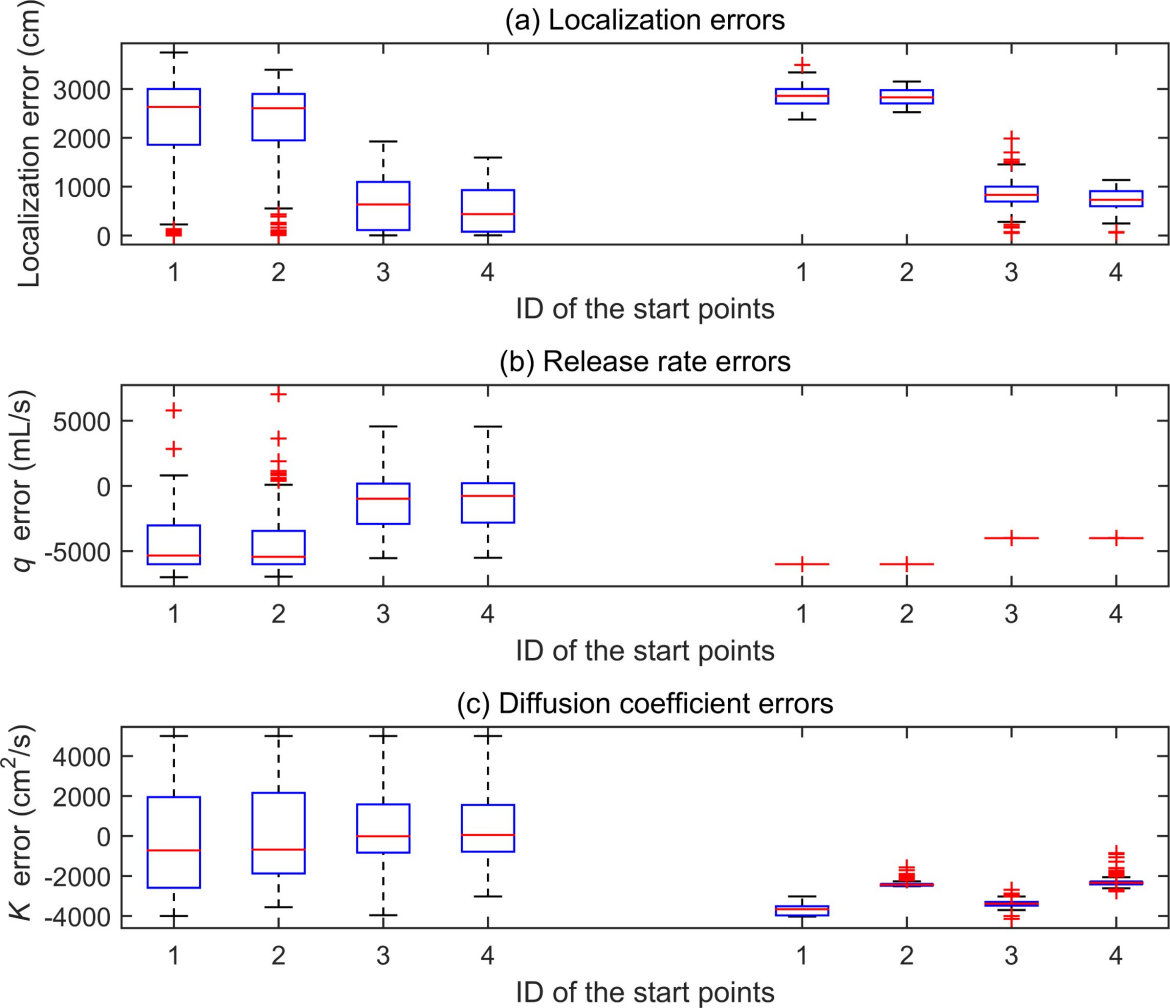
Boxplots of the estimation errors obtained by TRR and LM with respect to four different initial points. In each subfigure, the left four boxplots correspond with TRR, whereas the others correspond with LM.

Each of the four start points shown in Table 1 were tested for 200 times. The boxplots of the corresponding estimation errors are shown in Fig 5. In each subfigure of Fig 5, the left four and right four boxplots correspond to TRR and LM, respectively.

On one hand, the estimation errors in Fig 5 reveal that the estimation performance of LM and TRR were both obviously influenced by the start point. By utilizing the fourth start point, the LM and TRR both can achieve the smallest estimation errors. Possible reason is that, among all the start points, the fourth start point is closest to the real values.

On the other hand, it can be conceptually considered that the TRR is more likely to obtain small estimation errors than the LM algorithm. The TRR obtained a lot of small estimation errors. By contrast, the LM converged far from zero in most trails, although the results of the LM are more aggregated than those of the TRR.

### 4.2 Performance comparison

#### 4.2.1 PSPI with Gaussian measurement noises

**Fig 6:**
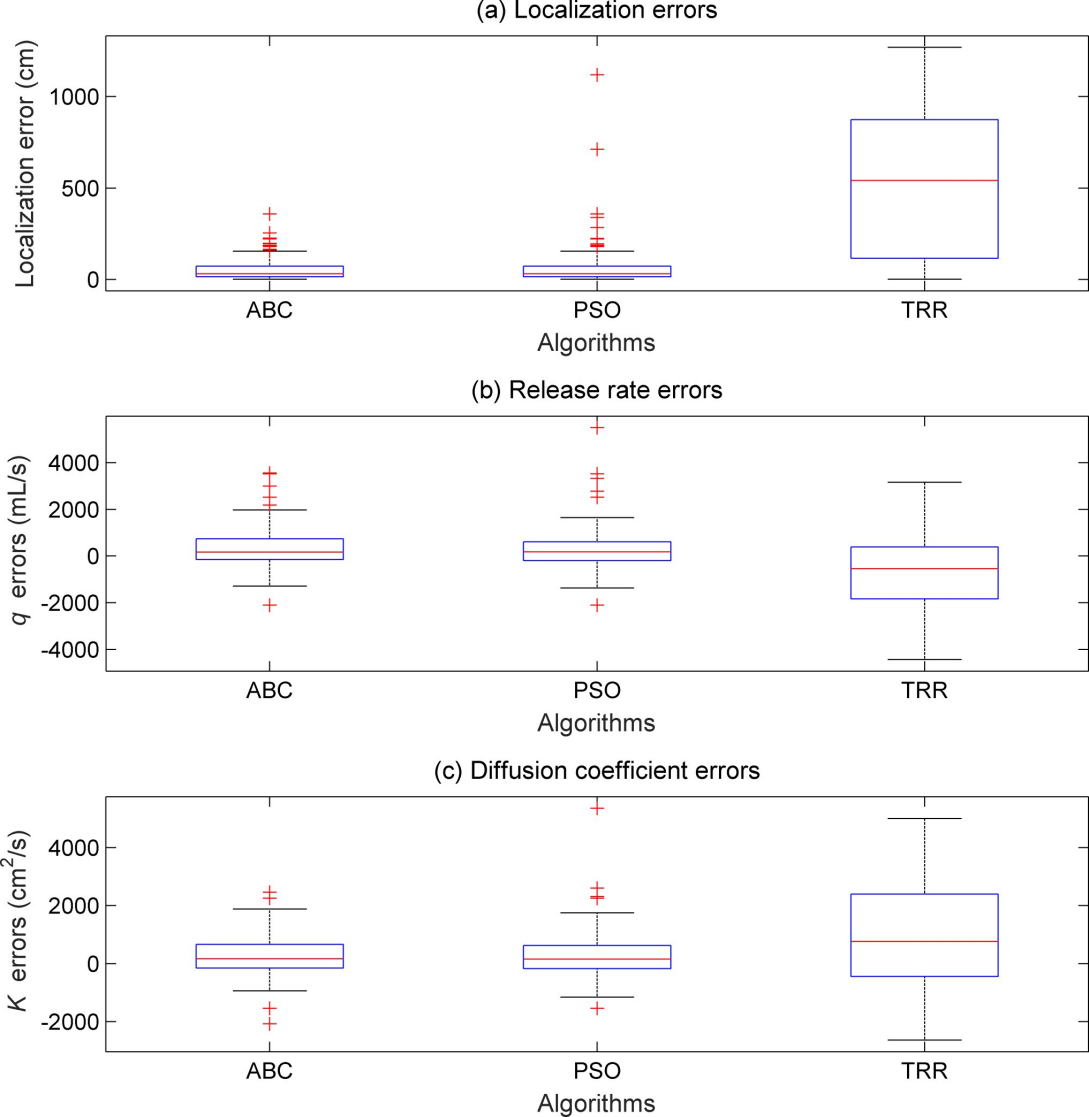
Boxplots of the estimation errors obtained by the ABC, PSO, and TRR.

On the basis of the parameter selection results in section 4.1, the WSN was randomly deployed for 100 times to compare the ABC with the PSO, TRR methods. For each of the random node deployment schemes, each of the ABC, PSO, TRR methods was tried once. For both ABC and the PSO, the population size and MCN were set as 100 and 500, respectively. The “*limit*” value of ABC was set as 1000. The inertial factor of PSO was set as 0.7. For the TRR, the start point was set as the fourth start point in Table 1. Other simulation setups are the same as those in section 4.1. The boxplots of the estimation errors are shown in Fig 6.

From Fig 6, we can see that the ABC and the PSO achieved similar estimation errors. This similarity is mainly because of the same measurement scheme and the similar search ability of the ABC and the PSO. Moreover, the IQR and estimation biases obtained by the TRR algorithm are much bigger than those of the ABC and the PSO. Note that the start point for the TRR algorithm has been intentionally set within the neighborhood of the real values (i.e., the fourth start point in Table 1), which is not applicable in real PSPI problems.

#### 4.2.2 PSPI with non-Gaussian measurement noises

**Fig 7:**
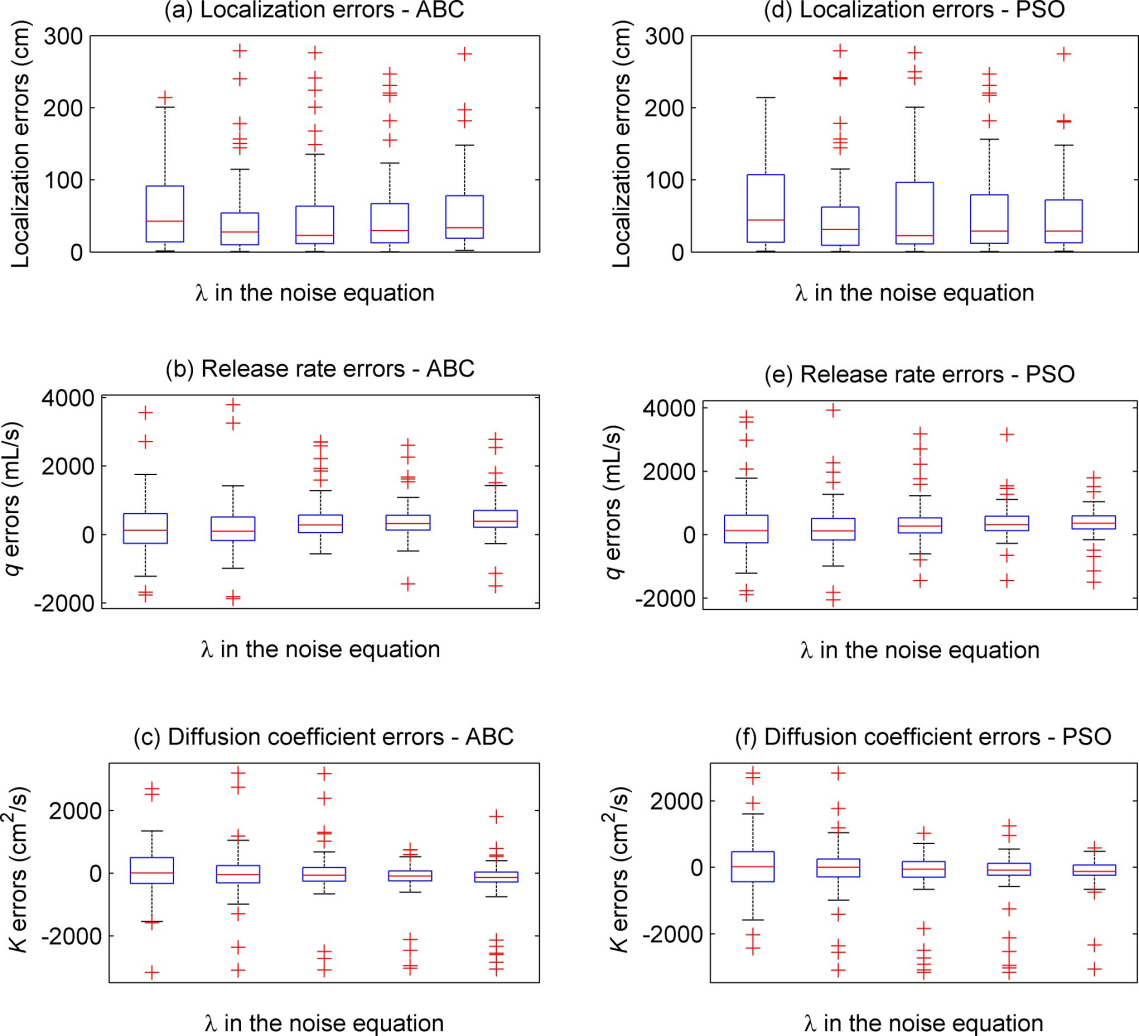
Boxplots of the estimation errors obtained by the PSO and ABC based on non-Gaussian measurement noises. In each subfigure, the left four boxplots correspond with the ABC, whereas others correspond with the PSO.

The above simulations are based on the assumption that the concentration measurement noises of the WSN satisfy the Gaussian distribution. However, this assumption is not valid in many applications with external interference factors. Thus, to test the algorithms’ robustness in respect of non-Gaussian measurement noises, measurement noises sampled from the contaminated Gaussian distribution function in Eq (4) were added into the concentration measurements of the WSN. The value of *λ* in Eq (4) were varied from 1 to 0 in intervals of 0.25. For each value of *λ*, the ABC and the PSO were both tried for 200 times. The boxplots of these simulations are shown in Fig 7. For better illustration, the display data ranges of Fig 7(A) and 7(D) were restrained as (0, 300) cm, which means a few outliers are not displayed in Fig 7(A) and 7(D).

As shown in the left four boxplots, the ABC obtained similar estimation errors when the value of *λ* varied. These estimation errors are generally close to zero. According to Eq (4), a smaller value of *λ* means that the Gaussian component takes less effect in the measurement noises. Therefore, the estimation performance obtained by the ABC does not rely on the distribution of measurement noise. In other words, the ABC is robust when it is used for solving the WSN-based PSPI problem. Moreover, the right four boxplots are similar as the left ones, which reveals that the PSO also achieved similar estimation results as those obtained by the ABC. This similarity corresponds with that demonstrated in Fig 6.

## 5. Conclusions

The ABC has been adapted for solving the WSN-based PSPI problem, in which the parameter values of a previously unknown pollution source should be identified based on the measurements of a wireless sensor network. According to the simulation results, the location, release rate, and diffusion coefficient of the pollution source can be simultaneously identified with considerable accuracy by using the ABC. Moreover, for solving the WSN-based PSPI problem, the ABC and the PSO obtained similar identification results in the same simulation scenario, although the ABC employs less tunable parameters than the PSO. As two intelligent optimization algorithms, the ABC and the PSO both obtained generally more accurate results than the TRR, which is a typical single-point search algorithm. The above conclusions are based on the result of computer simulations. Future works may cover collecting concentration measurements with a real WSN, and using them to evaluate the performance of the ABC and its counterpart algorithms.
